# Supplementary figures and images for: Innate defense mechanisms against Nosema ceranae in hygienic honey bee (Apis mellifera) colonies
Source: PLoS One. 2026 Mar 4;21(3):e0339548. doi: 10.1371/journal.pone.0339548 (PMC12959704; doi:10.1371/journal.pone.0339548)

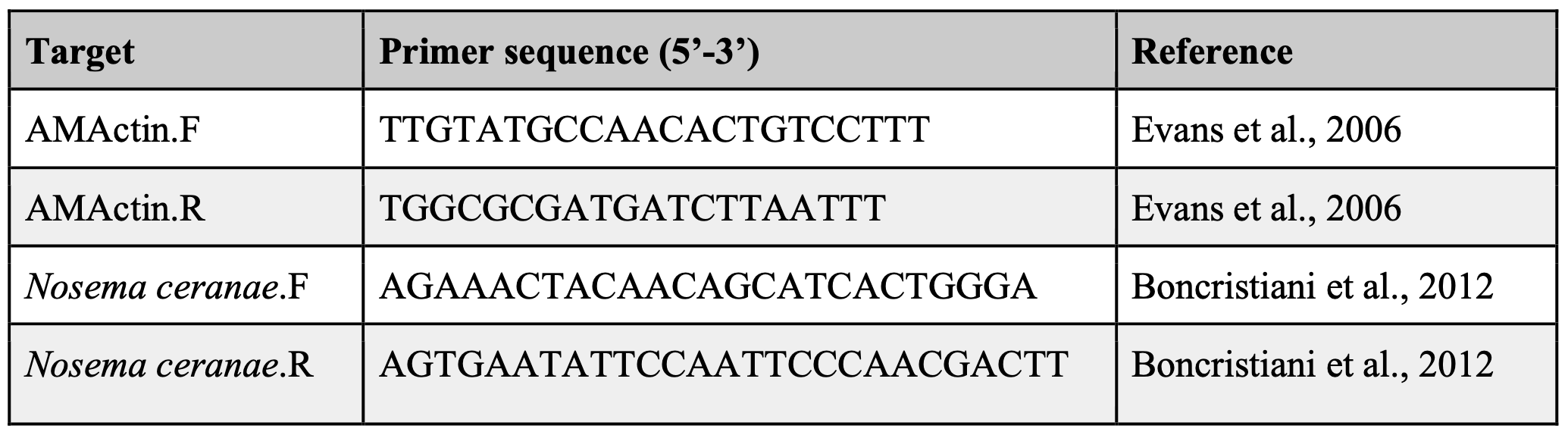

Supplement: S1 Table — (TIFF) [file pone.0339548.s001.tiff]

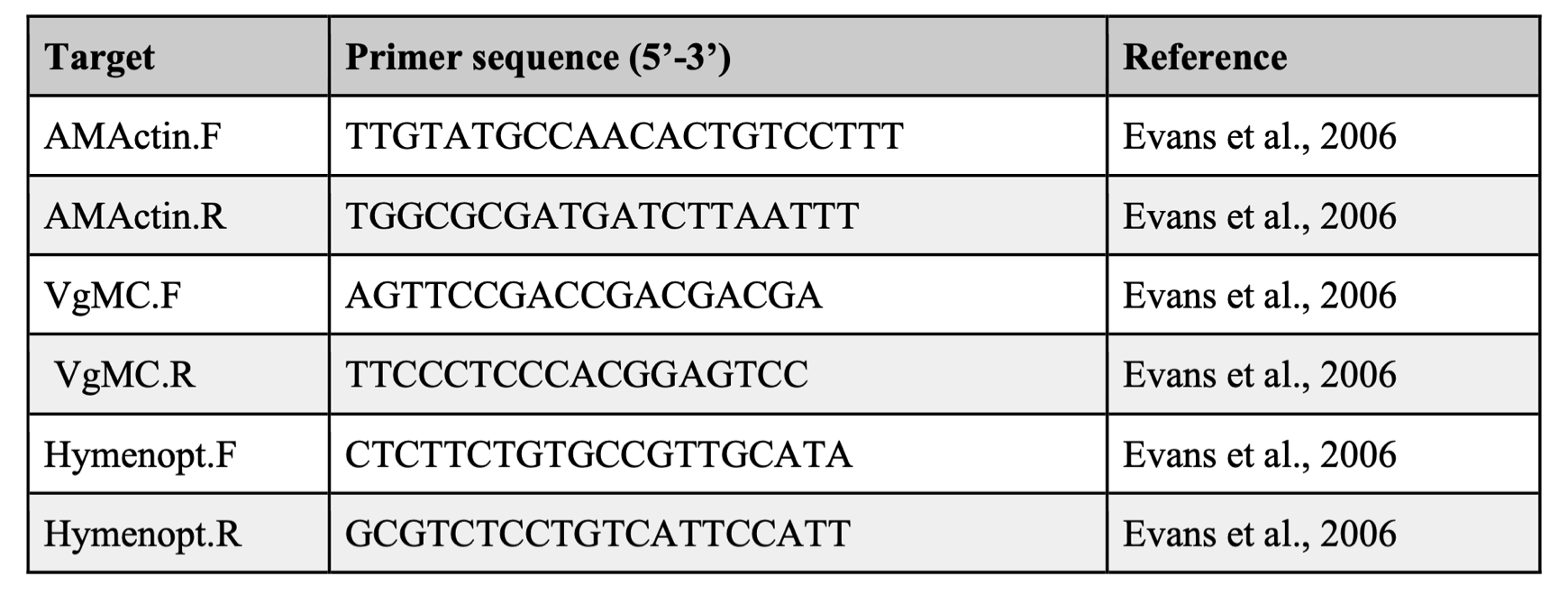

Supplement: S2 Table — (TIFF) [file pone.0339548.s002.tiff]

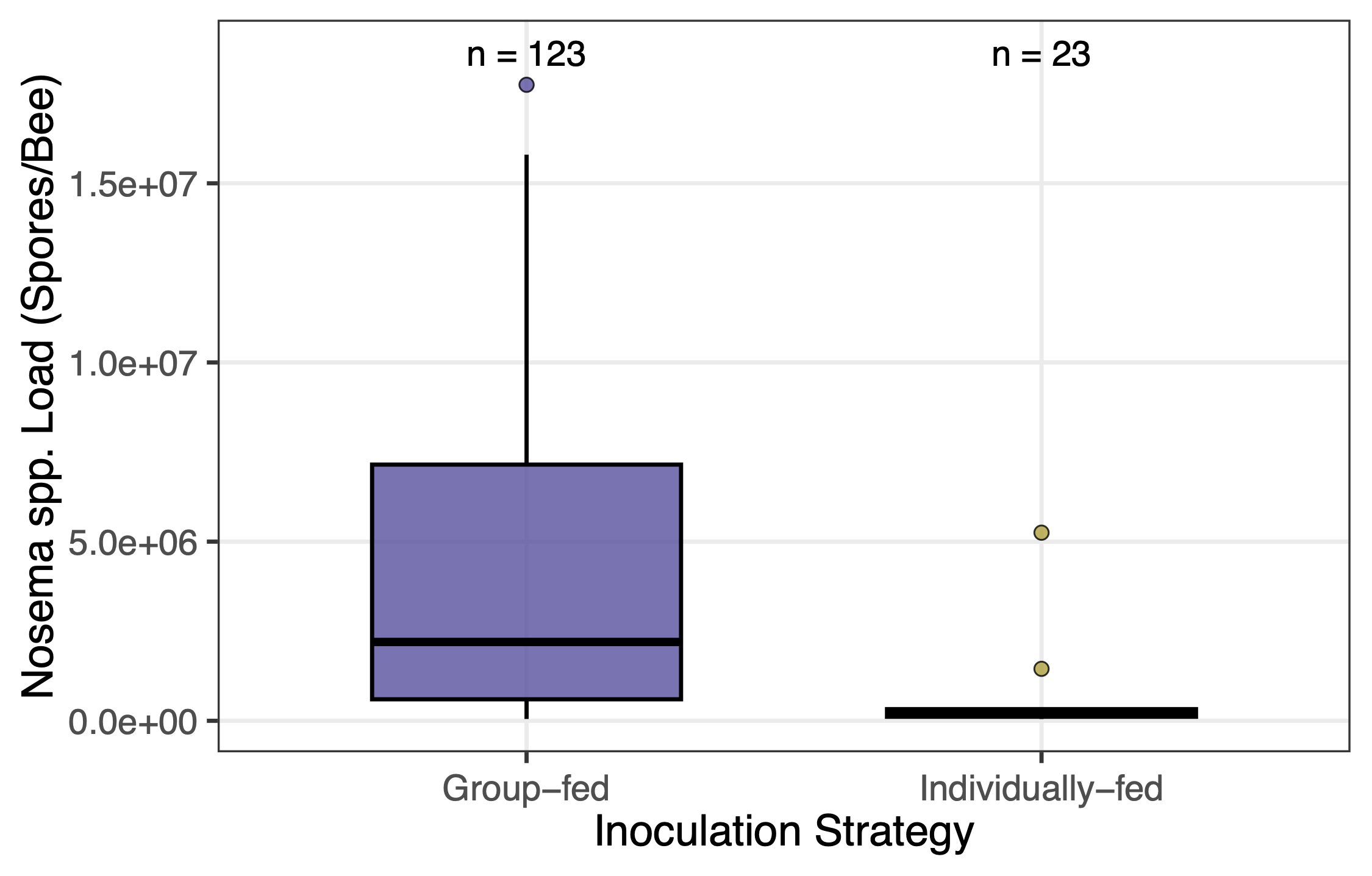

Supplement: S3 Fig — Nosema spp. loads (spores per bee) differed significantly between group-fed and individually fed bees (Welch’s t-test: t₃₅.₀₅ = 4.67, p < 0.001), with greater loads and variance among group-fed bees (Levene’s test: F₁,₈₁ = 5.52, p = 0.021). Purple boxes indicate group-fed bees, and yellow boxes indicate individually-fed bees. Sample sizes are denoted above each box. (TIFF) [file pone.0339548.s003.tiff]

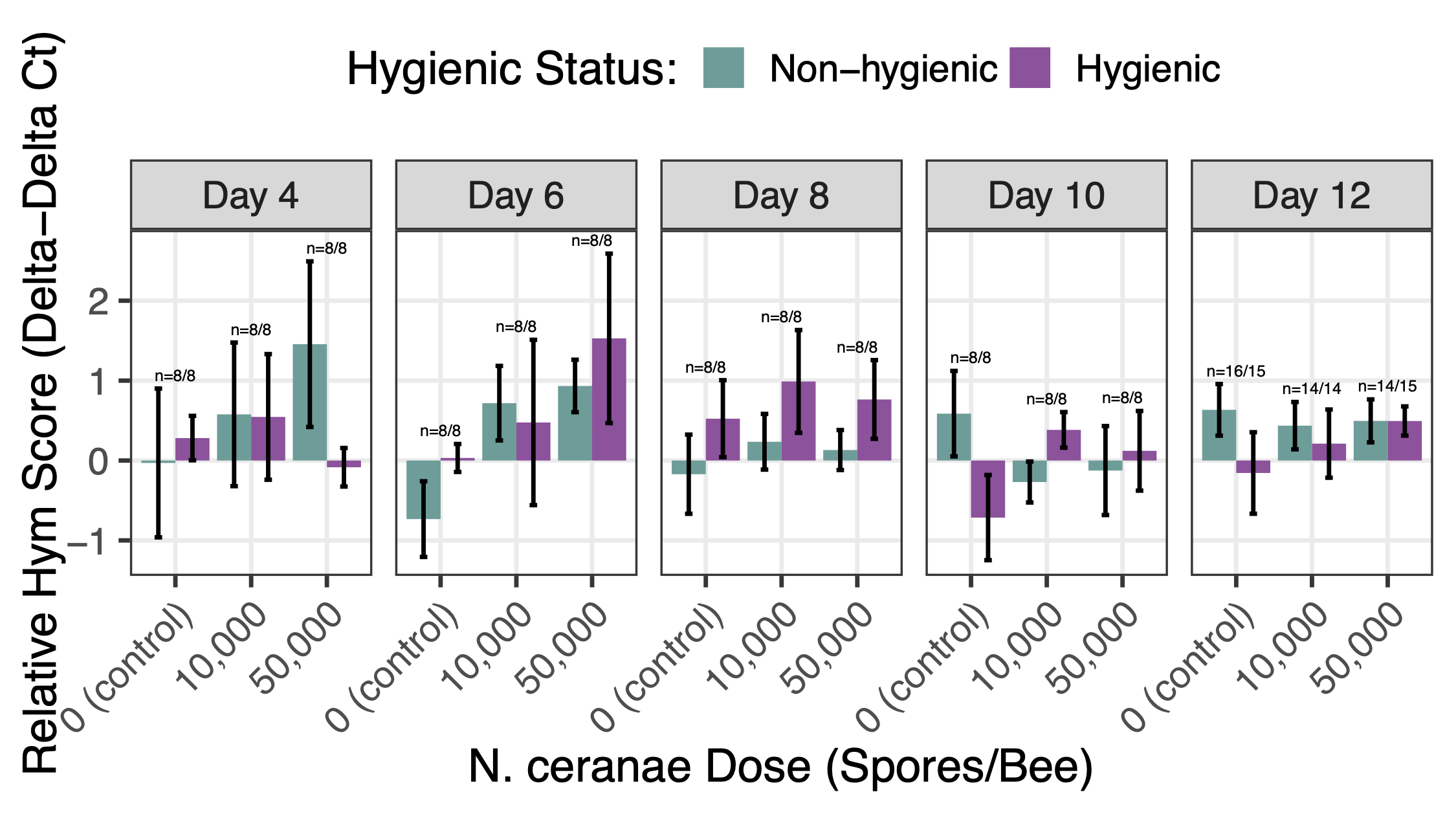

Supplement: S4 Fig — No significant main effects of colony hygienic status (χ²₁ = 0.012, p = 0.913), sampling day (χ²₄ = 3.33, p = 0.504), N. ceranae dose (χ²₂ = 3.23, p = 0.199), nor interaction effects between the predictor variables were detected. Hymenoptaecin expression levels are shown as the relative Hym score (ΔΔCt, log₁₀-transformed). Purple bars represent hygienic bees; green bars represent non-hygienic bees. Error bars represent standard errors of the mean. Sample sizes are denoted above each bar pair as n = hygienic bees/non-hygienic bees. Significance between pairs is denoted as ‘.’ < 0.1, ‘*’ < 0.05, ‘**’ < 0.01, ‘***’ < 0.001. (TIFF) [file pone.0339548.s004.tiff]
